# Regulation of *LYRM1* Gene Expression by Free Fatty Acids, Adipokines, and Rosiglitazone in 3T3-L1 Adipocytes

**DOI:** 10.1155/2012/820989

**Published:** 2011-10-26

**Authors:** Min Zhang, Hai-Ming Zhao, Zhen-Ying Qin, Rui Qin, Xiao-Hui Chen, Ya-Ping Zhao, Chun-Mei Zhang, Chun-Lin Gao, Chun Zhu, Chen-Bo Ji, Xin-Guo Cao, Xi-Rong Guo

**Affiliations:** ^1^Nanjing Maternal and Child Health Hospital of Nanjing Medical University, Nanjing 210004, China; ^2^Second Affiliated Hospital of Nanjing Medical University, Nanjing 210011, China; ^3^Institute of Pediatrics, Nanjing Medical University, Nanjing 210029, China; ^4^Jiangsu Maternal and Child Health Hospital of Nanjing Medical University, Nanjing 210036, China

## Abstract

LYR motif containing 1 (*LYRM1*) is a novel gene that is abundantly expressed in the adipose tissue of obese subjects and is involved in insulin resistance. In this study, free fatty acids (FFAs) and tumor necrosis factor-**α** (TNF-**α**) are shown to upregulate *LYRM1* mRNA expression in 3T3-L1 adipocytes. Conversely, resistin and rosiglitazone exert an inhibitory effect on *LYRM1* mRNA expression. These results suggest that the expression of *LYRM1* mRNA is affected by a variety of factors that are related to insulin sensitivity. *LYRM1* may be an important mediator in the development of obesity-related insulin resistance.

## 1. Introduction

Obesity has become a global public health problem in recent decades [1]. Type 2 diabetes is characterized by an inadequate beta-cell response to progressive insulin resistance, which is typically accompanied by weight gain [2]. The increasing global prevalence of type 2 diabetes is tied to rising rates of obesity [3]. Common obesity (complex polygenic obesity) results from interactions between genetic, environmental, and psychosocial factors [4]. However, the mechanisms underlying individual differences that lead to a predisposition to obesity remain obscure. 

In our earlier studies, we isolated and characterized LYR motif containing 1 (*LYRM1*), a novel human gene that was expressed at a high level in the omental adipose tissue of obese patients. *LYRM1* promotes preadipocyte proliferation and inhibits apoptosis of preadipocytes [5, 6]. Overexpression of *LYRM1* in 3T3-L1 adipocytes resulted in a reduction of insulin-stimulated glucose uptake, an abnormal mitochondrial morphology, decreased intracellular ATP synthesis, and decreased mitochondrial membrane potentials. In addition, *LYRM1* overexpression led to an excessive production of intracellular reactive oxygen species [7]. Our findings indicate that *LYRM1* may be a new candidate gene related to obesity-associated insulin resistance. 

Several studies have shown that adipose tissue in obese patients releases large amounts of free fatty acids (FFAs) and several adipokines, including tumor necrosis factor-*α* (TNF-*α*) and resistin [8–11]. All of these factors have been identified as major regulators of insulin activity. A synthetic activator of peroxisome proliferator-activated receptor-*γ* (PPAR-*γ*) called rosiglitazone (BRL49653) is part of the thiazolidinedione (TZD) class of drugs. Thiazolidinedione is one of a few classes of drugs that acts primarily as an insulin sensitizer by repressing, in mature adipocytes, the expression and secretion of adipokines [12]. However, the underlying molecular mechanisms of how these factors affect insulin sensitivity have not been clarified. 

In this study, we show that *LYRM1* is a novel gene related to obesity-associated insulin resistance. We hypothesize that these factors (FFAs, TNF-*α*, and resistin) and drug (rosiglitazone) may have a potential regulatory mechanism in obesity through the regulation of *LYRM1* mRNA expression, thereby affecting insulin sensitivity. The purpose of this study was to investigate the effects of FFAs, TNF-*α*, resistin, and rosiglitazone on *LYRM1* mRNA expression in 3T3-L1 adipocytes.

## 2. Materials and Methods

### 2.1. 3T3-L1 Cell Culture and Treatment

3T3-L1 cells were cultured, maintained, and differentiated as previously described [13]. Briefly, after confluence was achieved, the cells were grown for 2 days in DMEM/high-glucose medium (Gibco, Carlsbad, Calif, USA) supplemented with 10% fetal bovine serum (FBS; Gibco, Carlsbad, Calif, USA), in a 5% CO_2_ environment. Differentiation was subsequently induced by incubation in a similar medium that was supplemented with 0.5 mmol/L 3-isobuty-1-methylxanthine (MIX; Sigma, St. Louis, Mo, USA), 1 *μ*mol/L dexamethasone (Sigma, St. Louis, Mo, USA), and 10 *μ*g/mL insulin (Sigma, St. Louis, Mo, USA), for 2 days. The cells were then placed in a medium containing 10 *μ*g/mL insulin for another 2 days. Afterwards, the medium was replaced with DMEM containing only 10% FBS, every 2 days.

On the eighth day after differentiation was induced, if more than 90% of the cells showed the morphological and biochemical properties of adipocytes, the cells were used for experiments. After overnight incubation in serum-free DMEM, the 3T3-L1 adipocytes were treated with either 10 ng/mL TNF-*α* (T7539), 60 ng/mL resistin (SRP4560), 0.5 *μ*M rosiglitazone (375004), which were all dissolved in DMSO, or a 1 mM FFA cocktail composed of palmitic acid (p5585), oleic acid (O1008), and linoleic acid (L1376; Sigma, St. Louis, Mo, USA). The high FFA solution was prepared according to previously published methods [14, 15]. Briefly, the fatty acids were dissolved in 2% (w/v) fatty acid-free bovine serum albumin (BSA), with a stock concentration of 100 mM or an equivalent volume of vehicle. The stock solution was diluted 1 : 100 in DMEM to a final concentration of 1 mM. After 12 h or 24 h of incubation in the TNF-*α*, resistin rosiglitazone and high FFA solution, the adipocytes were collected for subsequent experiments.

### 2.2. Quantitative Real-Time Reverse Transcriptase-Polymerase Chain Reaction (RT-PCR)

Total RNA was extracted from 3T3-L1 adipocytes using Trizol reagent (Invitrogen, Carlsbad, Calif, USA). The extracted RNA was quantified by spectrophotometry at 260 nm. cDNA was synthesized from 1 *μ*g of total RNA using an AMV Reverse Transcriptase Kit (Promega A3500; Promega, Madison, Wis, USA), according to the manufacturer's instructions. Real-time RT-PCR was performed on an Applied Biosystems 7500 Sequence Detection System (ABI 7500 SDS; Foster City, Calif, USA) by following the manufacturer's protocol. 

Two primer sets were used for PCR analysis. A 259-bp DNA fragment within the *LYRM1* gene was used for the quantification of *LYRM1* mRNA. The PCR product had previously been cloned into the plasmid pMD-T 18 and verified by DNA sequencing. Plasmid standards of known copy numbers were used to generate a log-linear standard curve, from which the copy numbers of *LYRM1* could be determined by real-time qPCR. A 110-bp region of the **β*-actin* gene was used to normalize the results. A standard curve was generated from plasmids containing the **β*-actin* fragment. This standard curve was used to determine the copy numbers of **β*-actin*. Briefly, the samples were incubated at 95°C for 10 min for an initial denaturation, followed by 40 PCR cycles. Each cycle consisted of an incubation at 95°C for 15 s and annealing at 60°C for 1 min. The concentration ratio of *LYRM1* to **β*-actin* reflected the expression level of *LYRM1* mRNA per cell. Primer and Taqman probe (Invitrogen, Shanghai, China) sequences are shown in Table 1.

### 2.3. Statistical Analysis

Each experiment was performed at least three times. All data was expressed as means ± SD. Statistical analysis was performed using one-way ANOVA using the SPSS 12.0 statistical software package (SPSS Inc., Chicago, Ill, USA). For all tests, *P*-values less than 0.05 were considered statistically significant.

## 3. Results

### 3.1. The Expression of *LYRM1* mRNA during the Conversion of 3T3-L1 Preadipocytes into Adipocytes

**Figure 1:**
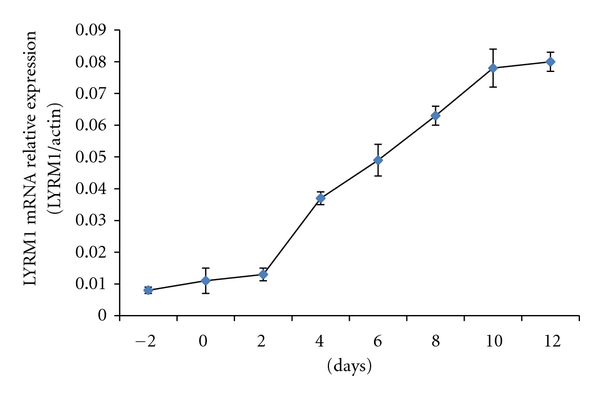
The expression of the *LYRM1* mRNA during the conversion of 3T3-L1 cells to adipocytes. 3T3-L1 cells were induced to differentiate, as described in “Materials and Methods” section. Total RNA was harvested from the 3T3-L1 cells on alternate days before (day −2, day 0) and after (day 2, day 4, day 6, day 8, day 10, and day 12) the switch from growth medium to differentiation medium. *LYRM1* mRNA levels were analyzed using quantitative real-time RT-PCR and normalized to **β*-actin* levels. The results are presented as the means ± SE of six experiments.

*LYRM1* mRNAs were expressed at very low levels In the 3T3-L1 preadipocytes. During the conversion of 3T3-L1 cells to adipocytes, the expression of the *LYRM1* gene was gradually increased to reach a stable level after the 10th day (Figure 1). More than 90% of the cells exhibited typical adipocyte morphology on the 10th day.

### 3.2. The Effect of FFAs on the Expression of *LYRM1* mRNA in 3T3-L1 Adipocytes

**Figure 2:**
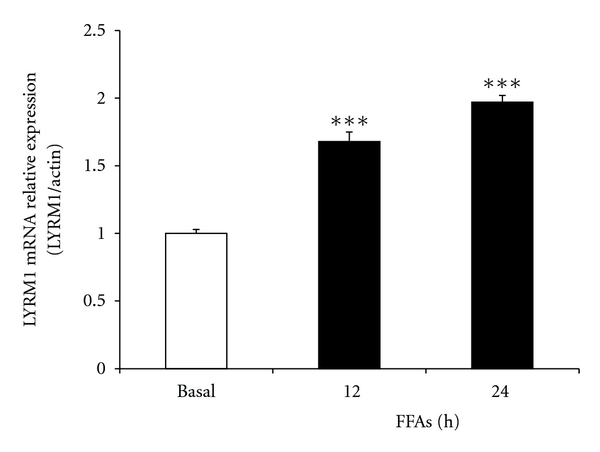
The effect of FFAs on the expression of *LYRM1* mRNA in 3T3-L1 adipocytes. Differentiated 3T3-L1 adipocytes were treated with 1 mM FFAs for the indicated periods (up to 24 h). *LYRM1* mRNA levels were analyzed using quantitative real-time RT-PCR and normalized to **β*-actin* levels. Results are presented as mean ± SE of six experiments. ***P < 0.001 in comparison with basal levels (untreated cells).

To assess the effect of FFAs on *LYRM1* mRNA levels, we examined the expression of *LYRM1* mRNA in 3T3-L1 adipocytes treated with 1 mM FFAs. Treatment durations were for either 12 or 24 h, 10 days after differentiation was stimulated. We found that FFAs concentrations of 1 mM led to a time-dependent increase in *LYRM1* mRNA expression. *LYRM1* mRNA expression dramatically increased after 12 h of exposure (Figure 2) and continued to increase after a 24 h exposure. At this time point, the expression of *LYRM1* mRNA was approximately 2-fold greater than the control mRNA (*P* < 0.001). This result shows that FFAs dramatically increased the mRNA expression level of the *LYRM1* gene.

### 3.3. The Effects of TNF-*α* and Resistin on the Expression of *LYRM1* mRNA in 3T3-L1 Adipocytes

**Figure 3:**
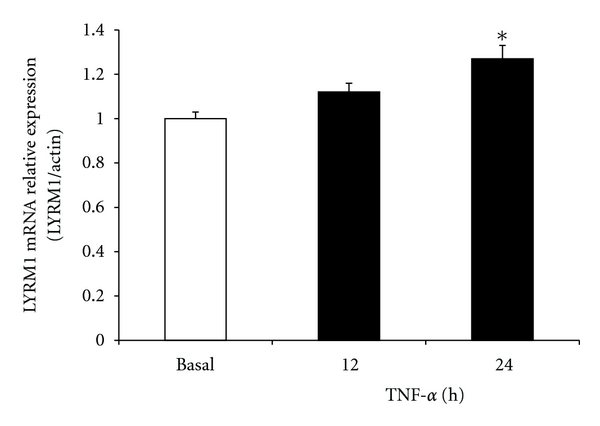
The effect of TNF-*α* on the expression of *LYRM1* mRNA in 3T3-L1 adipocytes. Differentiated 3T3-L1 adipocytes were treated with 10 ng/mL TNF-*α* for the indicated periods (up to 24 h). *LYRM1* mRNA levels were analyzed using quantitative real-time RT-PCR and normalized to **β*-actin* levels. Results are presented as mean ± SE of six experiments. *P < 0.05 in comparison with basal levels (untreated cells).

**Figure 4:**
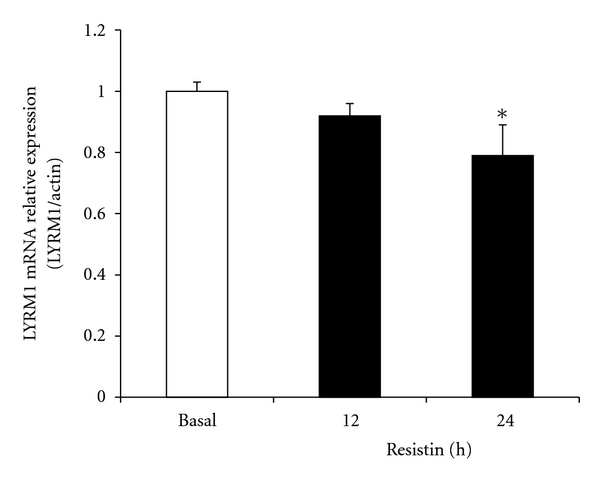
The effect of resistin on the expression of *LYRM1* mRNA in 3T3-L1 adipocytes. Differentiated 3T3-L1 adipocytes were treated with 60 ng/mL resistin for the indicated periods (up to 24 h). *LYRM1* mRNA levels were analyzed using quantitative real-time RT-PCR and normalized to **β*-actin* levels. Results are presented as mean ± SE of six experiments. *P < 0.05 in comparison with basal levels (untreated cells).

We examined *LYRM1* mRNA expression 10 days after differentiation was stimulated in 3T3-L1 adipocytes, which had been treated with 10 ng/mL TNF-*α* or 60 ng/mL resistin. TNF-*α* slightly increased *LYRM1* mRNA expression in 3T3-L1 adipocytes after 12 h. mRNA expression continued to increase 24 h after treatment (*P* < 0.05; Figure 3). Resistin showed a moderate inhibitory effect on *LYRM1* gene expression at 12 h; however, expression was significantly diminished 24 h after resistin treatment (*P* < 0.05; Figure 4).

### 3.4. The Effect of Rosiglitazone on the Expression of *LYRM1* mRNA in 3T3-L1 Adipocytes

**Figure 5:**
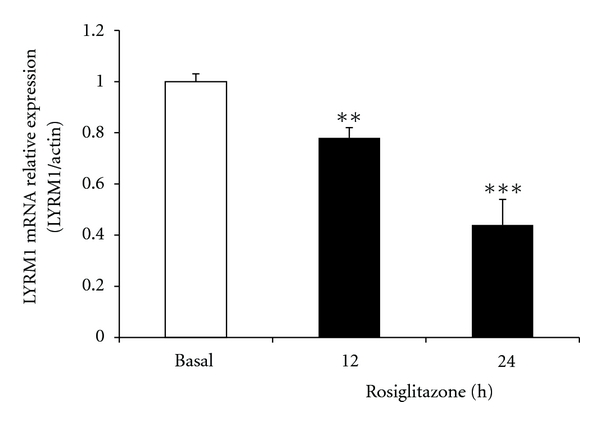
The effect of rosiglitazone on the expression of *LYRM1* mRNA in 3T3-L1 adipocytes. Differentiated 3T3-L1 adipocytes were treated with 0.5 *μ*M rosiglitazone for the indicated periods (up to 24 h). *LYRM1* mRNA levels were analyzed using quantitative real-time RT-PCR and normalized to **β*-actin* levels. Results are presented as mean ± SE of six experiments. **P < 0.01, ***P < 0.001 in comparison with basal levels (untreated cells).

To study the relationship between *LYRM1* expression and a PPAR-*γ* agonist, we examined the effect of rosiglitazone at 60 ng/mL on 3T3-L1 adipocytes. Twelve hours after treatment, *LYRM1* mRNA expression in 3T3-L1 adipocytes decreased. After 24 h mRNA expression had significantly diminished to approximately half that of the control (*P* < 0.001; Figure 5).

## 4. Discussion

The World Health Organization reports that at least one billion adults are overweight and 300 million are obese. In the absence of intervention, these numbers are expected to rise [16]. Most obese individuals are insulin resistant, which is an important etiological factor for type 2 diabetes mellitus. Adipocytes are known to secrete a variety of mediators, including FFA, TNF-*α*, and resistin, all of which regulate insulin signaling and glucose uptake. *LYRM1* is a recently discovered gene that is involved in obesity-associated insulin resistance [5, 7]. *LYRM1* mRNA expression is upregulated during conversion of 3T3-L1 cells to adipocytes, indicating that the expression of the *LYRM1* gene is involved in adipocyte differentiation. From the 10th day after induction of differentiation, the *LYRM1* mRNA expression remained at a stable high level, indicating that this clonal cell line can be used to investigate the regulation of *LYRM1* gene expression. To elucidate the mechanisms by which *LYRM1* is involved in the pathogenesis of obesity-associated insulin resistance, we characterized how this gene is regulated by factors that modulate insulin sensitivity. Furthermore, we also investigated the effects of rosiglitazone, which is a PPAR-*γ* agonist, on *LYRM1* mRNA expression in 3T3-L1 adipocytes. 

Elevated concentrations of circulating free fatty acids are characteristic of type 2 diabetes and are implicated in the etiology of insulin resistance [17]. Insulin resistance is thought to arise from impaired insulin signaling in target tissues. Signaling is impaired due to augmentation of the serine/threonine phosphorylation sites of insulin receptor substrates (IRS-1 and IRS-2). In addition, insulin resistance is compounded by a reduction of activated PI3-kinase (PI3K) and an inhibition in the translocation of insulin-stimulated glucose transporter 4 (GLUT4) [18, 19]. An excess of FFAs causes the intracellular accumulation of metabolic products such as ceramides, diacylglycerol, or acyl-CoA. These FFA-derived products may lead to defects in insulin signaling and glucose transport through the PI3K-dependent pathway [20, 21]. However, the underlying mechanisms of these phenomena have not been clarified. In this study, we observed that FFAs added exogenously upregulated *LYRM1* mRNA expression in 3T3-L1 adipocytes. We had previously shown that *LYRM1* overexpression can inhibit insulin-stimulated glucose transport in adipocytes [7]. We observed that an excess of FFAs might induce insulin resistance. Resistance could partly be induced through the upregulation of *LYRM1* expression, which would inhibit glucose uptake in adipocytes. These findings support and extend other results in the literature that investigate the effects of FFAs on insulin signaling.

As one of the most widely studied cytokines, TNF-*α* is reported to modulate insulin resistance [10]. A key role for TNF-*α* in obesity-related insulin resistance was identified when TNF-*α* or TNF-*α* receptors were deleted in both diet-induced obese mice and leptin-deficient ob/ob mice, which resulted in significantly improved insulin sensitivity [22]. However, the infusion of TNF-*α*-neutralizing antibodies into obese, insulin-resistant subjects, or type 2 diabetic patients, did not improve insulin sensitivity [23, 24]. In this study, we observed that TNF-*α* slightly upregulates *LYRM1* mRNA expression in 3T3-L1 adipocytes. There is a need for further studies in human adipocytes. Currently, we suggest that TNF-*α*-induced insulin resistance is only indirectly involved in increased *LYRM1* expression. 

*Resistin* was identified as a gene that was downregulated by TZD in mouse adipocytes [11]. In rodents, the circulating levels of resistin increased in obesity [25]. Furthermore, an increase in serum resistin levels induced insulin resistance in several rat and mouse models, including after acute administration [26]. Recombinant *resistin* caused severe hepatic insulin resistance in rodents [26]. However, a study observed a decrease in fasting glucose, improved glucose tolerance and enhanced insulin sensitivity in resistin knockout mice [27]. In humans, there is considerable controversy surrounding the role of *resistin*. We showed that resistin exerts a moderate inhibitory effect on *LYRM1* gene expression in 3T3-L1 adipocytes. This data suggests that *LYRM1* and resistin interact during the development of obesity-associated insulin resistance.

In this study, we observed that rosiglitazone inhibits *LYRM1* gene expression in 3T3-L1 adipocytes. Rosiglitazone is part of the TZD class of drugs, which act as insulin sensitizers and agonists for the transcription factor PPAR-*γ*. PPAR-*γ* is a member of three nuclear receptor isoforms (the other two are PPAR-*α* and PPAR-*δ*), which are encoded by different genes. PPAR-*γ* is the master regulator of adipogenesis, being both essential and sufficient for adipocyte differentiation [28]. It also upregulates the expression of fatty acid transporter proteins (FATP-1 and D036) [29]. Rosiglitazone suppresses TNF-*α* mediated inhibition of adipocyte differentiation, whilst TNF-*α* decreased the expression of PPAR-*γ* [30]. TZDs inhibit resistin gene expression in human macrophages [31, 32] and lower serum resistin levels in humans as well as rodents [33–35]. We deduced that rosiglitazone inhibits *LYRM1* gene expression most likely through PPAR-*γ*. 

Our results demonstrate that *LYRM1* mRNA expression is greatly affected by rosiglitazone, FFAs, and two adipokines, TNF-*α* and resistin. These two adipokines are involved in the regulation of insulin sensitivity. The upregulation or downregulation of *LYRM1* expression may be strongly linked to FFA or rosiglitazone-related insulin resistance. Recently, *LYRM1* in rat myoblasts has been shown to negatively regulate the function of IRS-1 and PI3K/Akt, whilst decreasing GLUT4 translocation and glucose uptake in response to insulin (L6) [36]. However, a more precise characterization of the physiological activities of *LYRM1* is required to fully understand these processes.

## Figures and Tables

**Table 1:** Nucleotide sequences for primer and probe sets used in qPCR.

Gene	Forward primer (5′–3′)	Probe	Reverse primer (5′–3′)
*LYRM1*	CAGATGGATAGGGCGTGGATAAGG	TGGTAATGCAGTCCAATCTCAATCCG	GACAGCAGCAACCCGACAAGAAGT
**β*-actin*	CCTGAGGCTCTTTTCCAGCC	TCCTTCTTGGGTATGGAATCCTGTGGC	TAGAGGTCTTTACGGATGTCAACGT
